# Potential negative effects of artificial intelligence in Kazakhstan’s public sector: an analysis of hidden risks

**DOI:** 10.3389/frai.2026.1781993

**Published:** 2026-05-25

**Authors:** Malika Buribayeva, Zhanna Khamzina, Yermek Buribayev

**Affiliations:** 1 Department of Computer Science and Engineering, Lehigh University, Bethlehem, PA, United States; 2 Department of Law, Zhetysu University named after I. Zhansugurov, Taldykorgan, Kazakhstan

**Keywords:** algorithmic governance, artificial intelligence, digital state, hidden risks, public administration, regulatory policy

## Abstract

**Introduction:**

This article presents an in-depth single-case study of Kazakhstan and examines the latent negative effects that may accompany the expanding use of artificial intelligence (AI) in the public sector. The central premise is that such risks arise less from isolated technical performance indicators of particular AI systems than from the broader architecture of legal regulation, institutional design, and centralized data infrastructures.

**Methods:**

The study combines normative-institutional analysis with secondary qualitative analysis. The normative component examines the Law of the Republic of Kazakhstan “On Artificial Intelligence,” the National AI Platform, and related digital governance projects, including Smart Data Ukimet, the Digital Family Card, and sovereign large language models. The empirical component is based on a secondary qualitative analysis of 53 semi-structured interviews with civil servants. Auxiliary analytical summaries derived from the same interview subset were used only to support retrieval and thematic consolidation. The analysis therefore reconstructs primarily ex ante and design-level risk trajectories rather than providing a full ex post assessment of realized harms, although some enabling mechanisms are already observable in existing digital infrastructures.

**Results:**

On this basis, the article develops a map of hidden AI risks adapted to the Kazakhstani context. Five main groups of risks are distinguished: political-legal and institutional risks; data- and technology-related risks; organizational and managerial risks; explainability and opacity risks; and market and environmental risks. The findings show that, in the Kazakhstani case, these risks are institutionalized ex ante through legal constructs, the distribution of administrative powers, and established data-use practices. AI is legally framed as a “tool,” while a de facto redistribution of power occurs through algorithmic and infrastructural arrangements. Centralized data infrastructures, including Smart Data Ukimet, the Digital Family Card, and the National AI Platform, may intensify algorithmic stratification and systemic bias. In addition, the mass deployment of AI agents under KPI-based governance may contribute to deskilling and to the fragmentation of responsibility among developers, public agencies, and citizens.

**Discussion:**

The article argues that the principal challenge of AI deployment in Kazakhstan’s public sector lies not only in the accuracy, efficiency, or technical reliability of individual systems, but also in the institutional conditions under which these systems are embedded. The Kazakhstani case demonstrates how AI-related risks can be formed before large-scale harms become empirically visible, through regulatory definitions, centralized infrastructures, and governance models that redistribute authority while obscuring responsibility. The study contributes to debates on public-sector AI governance by emphasizing the need for anticipatory institutional safeguards, transparent accountability mechanisms, and stronger scrutiny of data infrastructures in highly centralized administrative contexts.

## 1 Introduction

In recent years, artificial intelligence has become an integral element of the contemporary architecture of public administration. Unlike previous waves of digitalization, which were primarily oriented toward the automation of document circulation and the development of electronic services, the deployment of AI affects the very logic of public decision-making and leads to a redistribution of roles among humans, data, and algorithms. The state, traditionally understood as an institution that makes decisions on the basis of formalized procedures and expert judgment, increasingly functions as an operator of complex socio-technical systems within which AI performs the functions of filtering, evaluation, forecasting, and allocation.

AI adoption in government is commonly justified by its potential to improve service quality, speed, and consistency, especially in high-volume administrative processes. At the same time, international experience shows that AI can generate negative externalities, including unequal treatment, opacity, goal displacement, and new forms of infrastructural dependence, when deployed without adequate safeguards. The present article therefore focuses on risks not to reproduce a “doom and gloom” narrative, but to provide an analytically grounded assessment of vulnerabilities that may be overlooked in official modernization discourse.

The starting hypothesis of the study is that the most significant risks of AI in the public sector are determined less by isolated technical metrics of individual models (e.g., accuracy) and more by the way the state constructs the legal regime, institutional architecture, and data infrastructures within which AI systems are trained, deployed, and governed. In particular, bias is treated not as a property of an “algorithm as such,” but as an outcome of the data, objectives, and institutional arrangements that shape model behavior. Accordingly, the focus of analysis is placed on AI as an element of the configuration of Kazakhstan’s digital state, rather than as a purely technical artifact detached from context.

In this respect, Kazakhstan represents an illustrative example of a state in which the course toward AI development has acquired an explicitly articulated normative-institutional design. The Concept for the Development of Artificial Intelligence for 2024–2029 was approved by Government Resolution No. 592 (24 July 2024) (Government of the Republic of Kazakhstan, 2024), and the institutional architecture was further centralized through the creation of the Ministry of Artificial Intelligence and Digital Development (hereafter, the Ministry) by Presidential Decree No. 997 (18 September 2025) (President of the Republic of Kazakhstan, 2025). A dedicated Law of the Republic of Kazakhstan “On Artificial Intelligence” (No. 230-VIII, adopted 17 November 2025; entered into force 18 January 2026) introduced a risk-based logic of AI governance by classifying systems by risk and specifying prohibited practices, a logic broadly consonant with the EU’s AI Act (Law of the Republic of Kazakhstan, 2025; Regulation (EU) 2024/1689, 2024; International Center for Not-for-profit Law, 2025). Taken together, these instruments frame AI not only as a tool of administrative modernization, but also as an object of targeted state policy embedded in strategic planning and sectoral regulation.

Empirically, AI in Kazakhstan’s public sector is operationalized through the eGov ecosystem and integrated data infrastructures that enable automated eligibility checks, risk scoring, and proactive service delivery. Two emblematic examples are Smart Data Ukimet, an information-analytical platform designed to consolidate and analyze data from more than 120 governmental databases, and the Digital Family Card, which aggregates registry data to profile household welfare and to trigger proactive social services and notifications (National Information Technologies JSC, n.d.; UNDP Kazakhstan, 2024). The emerging ecosystem also includes the National AI Platform, sovereign large language models, and AI assistants and agents being introduced into e-government and administrative workflows (Madiyev, 2025). Taken together, these infrastructures and interfaces pre-structure how AI and analytics are implemented across agencies and therefore constitute a key locus for systemic, architecture-level risks.

At the same time, such accelerated institutionalization of the AI agenda gives rise to a number of fundamental questions. First, how the principles of legality, fairness, transparency, explainability, the priority of human well-being, data protection, and confidentiality declared in legislation and strategic documents are translated into operational mechanisms of responsibility and accountability within specific administrative processes. Second, how the concentration of key roles of AI development, operation, regulation, and promotion within a limited circle of state and quasi-state actors affects the configuration of risks. Third, what consequences follow from the formation of a centralized infrastructure of data and computing capacities that serves both public and commercial AI applications.

Despite the expanding literature on AI governance and on the “dark side” of AI in public administration, research remains limited on how risk-based AI legislation, centralized data infrastructures, and national platforms jointly “bake in” vulnerabilities in post-Soviet digital states. Moreover, existing work on Kazakhstan tends to separate doctrinal legal analysis from the infrastructural and organizational mechanisms through which public services are actually delivered, and it rarely combines normative-institutional analysis with structured qualitative evidence on how civil servants perceive emerging digital risks. By bringing these dimensions together through a normative-institutional analysis and a secondary qualitative analysis of 53 semi-structured interviews with civil servants, the article aims to fill this gap and to develop a context-specific map of latent, or hidden, AI risks.

To provide an analytical response to these questions, the article introduces the category of “hidden” (latent) AI risks in the public sector. Here, hidden risks are understood as potential negative effects that remain weakly articulated in official discourse as governance problems, are not reducible to defects of individual AI models, and are primarily produced by the institutional and infrastructural characteristics of the digital state. Given that Kazakhstan’s AI Law entered into force in January 2026, the analysis is predominantly ex ante. It maps vulnerabilities embedded in design choices prior to, or in the early stages of, large-scale deployment. At the same time, because centralized data infrastructures and proactive service architectures are already operational, some of these mechanisms may already be observable in practice even before AI systems are deployed at full scale.

The article treats these risks primarily as emergent structural effects of legal design, infrastructural centralization, and administrative routines rather than as evidence of a centrally coordinated strategy of AI-enabled social control. Where the analysis refers to epistemic steering, asymmetrical visibility, or the narrowing of contestable interpretations, the claim is that such outcomes may arise from the institutional configuration of public-sector AI even in the absence of explicit coercive intent.

This category may include, inter alia, the redistribution of power in favor of data and technology operators, the displacement of the locus of accountability from humans to the “system,” the structural entrenchment of discriminatory practices within algorithmic procedures for the allocation of resources and services, as well as the normalization of opacity and the de facto inaccessibility of algorithmic decisions to external oversight.

The present study relies on a socio-technical understanding of AI, treating it as an assemblage of models, data, infrastructures, institutions, and practices. It also draws on conceptual frameworks developed in the international debate on the “dark side” of AI in public administration. This socio-technical perspective is specified in Section 2.1.

The purpose of the paper is to identify and describe hidden risks associated with the use of AI in Kazakhstan’s public sector and to demonstrate how these risks can be entrenched at the level of legal, institutional, and infrastructural design.

Based on the outlined problem field, the main research question is formulated as follows: which types of potentially negative consequences of AI use in Kazakhstan’s public administration are determined primarily by the architecture of regulation, institutions, and data infrastructures, rather than by isolated technical properties of AI systems.

Three subsidiary research questions guide the analysis: (i) how do the principles declared in the AI Law (legality, fairness and equality, transparency and explainability, the priority of human well-being, data protection, and confidentiality) translate into operational mechanisms of responsibility and accountability; (ii) which risk categories appear most salient for the current stage of Kazakhstan’s digitalization (eGov, Smart Data Ukimet, Digital Family Card, the National AI Platform, and related solutions); and (iii) which risks remain latent, weakly articulated in official discourse and doctrine, yet are already embedded in the normative-institutional design.

The research hypothesis is that latent AI risks in Kazakhstan are being institutionalized less through isolated technical properties of models than through three design features of the emerging AI regime: the concentration of data and algorithmic capacity in state and quasi-state hands, the under-operationalization of safeguards for control and risk management, and the concentration within the Ministry of regulatory, infrastructural, and promotional roles. On this basis, the article develops a five-block map of hidden AI risks in Kazakhstan’s public sector, aligned with international socio-technical typologies.

## 2 Literature review and conceptual framework

Section 2 reviews the scholarship most directly relevant to hidden AI risks in public administration. It begins by specifying the socio-technical premise adopted in this article (Section 2.1). It then examines five strands of prior research corresponding to the five domains of risk analyzed below: political-legal and institutional arrangements; data infrastructures; organizational and managerial transformation; explainability and opacity; and platform governance and environmental externalities (Sections 2.2.1–2.2.5). The section concludes by identifying the Kazakhstan/CIS research gap and the specific contribution of the present article (Section 2.3). Methodological self-positioning regarding the normative-institutional analysis and the single-case design is reserved for Section 3.

### 2.1 Socio-technical AI and hidden/systemic risks

A socio-technical perspective treats AI in public administration not as a standalone algorithmic artifact, but as a configuration that couples models and data with organizational routines, legal mandates, infrastructures, and the politics of classification and measurement. This literature emphasizes that the effects of AI systems are co-produced by technical design and institutional context and therefore cannot be assessed solely through model-centric metrics (Orlikowski and Iacono, 2001; Star and Ruhleder, 1996).

In this article, “hidden” risks refer to systemic vulnerabilities that arise at the level of infrastructures and governance arrangements, including how data are assembled, categories stabilized, and accountability distributed across interdependent actors. Such risks may remain latent because they are embedded in background infrastructures and standard operating procedures rather than recognized as discrete failures (Bowker and Star, 1999; Kitchin, 2017). This conceptual starting point directs attention away from isolated model outputs and toward the legal, institutional, and infrastructural conditions under which AI is introduced into public administration.

### 2.2 Main strands of scholarship on AI risks in public administration

The literature most relevant to the present study can be grouped into five interrelated strands. Together, these strands show that AI-related harms in government are generated not only by model properties, but also by the architecture of law, data infrastructures, organizational routines, explainability regimes, and platform-level externalities.

#### 2.2.1 Political-legal and institutional dimensions

The first strand of scholarship conceptualizes the “dark side” of AI in government primarily in political, legal, and institutional terms. Valle-Cruz et al. (2023) and Wirtz et al. (2020, 2022) develop socio-technical frameworks that relate AI risks in public administration to shifts in power, legal and regulatory gaps, accountability deficits, and vulnerabilities rooted in data and technological infrastructures, arguing that the most consequential harms stem from the institutional settings in which algorithmic systems are authorized and deployed rather than from isolated technical failures.

Building on these foundations, a growing body of scholarship in CIS and neighboring jurisdictions examines the regulatory forms through which such risks are mediated. This literature compares risk-based, technology-driven, and hybrid approaches to AI regulation; identifies unresolved questions in liability allocation, stakeholder definition, and procedural accountability; and warns against an “imperfect law syndrome,” in which rapidly proliferating but fragmented legislation fails to secure effective transparency and accountability of AI systems in the public sector (Kuteynikov et al., 2022; Tereschenko and Tokolov, 2025; Erahtina, 2023; Furashev and Korzh, 2025; Alekseeva, 2025; Rassolov and Chubukova, 2022). In the Kazakhstani and broader CIS context, legal-doctrinal and sector-specific studies have mapped emerging AI policies and applications in public administration, law, medicine, arbitration, and related domains, highlighting fragmented regulation, underdeveloped safeguards for human rights and privacy, and unresolved questions of accountability and professional responsibility (Konusova, 2023; Zhaltyrbayeva, 2023; Aubakirova and Moldabekov, 2024; Dauletkhanova, 2022; Nurakhmetova et al., 2024; Chernyh, 2020; Gracheva and Aryamov, 2020; Dudnichenko, 2024; Afanas’ev and Afanas’eva, 2025). Complementary social, ethical, and criminological analyses further show that institutional and legal arrangements condition broader harms such as structural unemployment, economic inequality, cybercrime, and privacy violations (Aleshkovski, 2022; Donika, 2023; Budko, 2024). Taken together, this strand shows that AI risks in digital states are inseparable from the allocation of authority, the distribution of accountability, and the completeness of legal safeguards.

#### 2.2.2 Data infrastructures and integrated information systems

A second strand approaches AI risks through the lens of data infrastructures and integrated information systems. Studies on digital transformation in public administration show that government agencies operate under specific constraints regarding sensitive data, legal mandates, and data-reuse rules, which shape the trajectories of AI adoption in ways that differ from the private sector (Blinova et al., 2021). Research on AI readiness in public administration further demonstrates that fragmented data architectures, legal and regulatory gaps, misaligned decision-making processes, and inefficient budget allocations can institutionalize risks long before sophisticated AI systems are widely deployed (Stupin, 2024; Nuruly et al., 2025). In parallel, the emerging literature on data governance and digital platforms conceptualizes public-sector data as a form of infrastructure. It highlights how state-sponsored data spaces, interoperability frameworks, and platform architectures may generate new market failures and power asymmetries if access rules, interoperability standards, and accountability mechanisms are weak or under-specified (Ducuing, 2020; Ducuing and Reich, 2023; Montero and Finger, 2021; Krarup and Horst, 2023; Prug and Bilić, 2024). These contributions underscore that the configuration of data infrastructures is not a neutral technical choice but a key determinant of how AI-driven risks are distributed and institutionalized. This issue is especially salient for languages such as Kazakh, where recent work on language models highlights scarce high-quality corpora, morphological richness, and the need for tailored tokenization, parameter-efficient fine-tuning, and retrieval-augmented or domain-adapted workflows rather than simple transplantation of English-centric models (Kadyrbek et al., 2025; Mansurova et al., 2025; Tukeyev et al., 2026).

#### 2.2.3 Organizational and managerial effects

A third strand examines how AI and broader digitalization reshape organizational structures, professional roles, and managerial practices in the public sector. Analyses of “rational bureaucracy 2.0” argue that digital tools increase external visibility and auditability of bureaucratic behavior while also embedding new forms of control, metric-driven performance management, and path-dependent organizational change (Blinova et al., 2021; Buklemishev, 2022; Bezvikonnaya, 2025). The AI Readiness Level index developed for Russian public administration illustrates how AI projects interact with existing institutional routines and budgetary processes, showing that risk management often remains implicit and fragmented (Stupin, 2024). In Kazakhstan and other CIS settings, sector-specific studies highlight that AI is introduced into environments already marked by hierarchical structures, formalism, digital inequality, and skill gaps, raising concerns over job displacement, deskilling, and the erosion of critical thinking in education and public employment (Mukhiyayeva et al., 2025; Askarkyzy and Zhunusbekova, 2024). This organizational perspective is directly relevant to the displacement of expertise, the fragmentation of accountability, and the normalization of metricized control in public bureaucracies.

#### 2.2.4 Explainability and opacity

A fourth strand focuses on transparency, explainability, and opacity in algorithmic governance. Within Russian-language legal scholarship, Kabytov and Nazarov conceptualize the “black box” problem as a core challenge that undermines trust in state institutions and access to judicial protection, stressing information asymmetries between citizens, public authorities, and private contractors, tensions between transparency requirements and the protection of trade secrets, and the high cost of implementing explainability mechanisms (Kabytov and Nazarov, 2025). Atabekov, in turn, shows how technical architectures of AI systems can acquire an implicit regulatory character, hard-wiring negative effects into governance arrangements when gaps in transparency, responsibility, and compliance persist (Atabekov, 2023). Internationally, a broader literature on algorithmic transparency and explainable AI discusses how different types of explanations, disclosure requirements, and audit regimes affect perceived fairness, accountability, and trustworthiness of algorithmic decisions in government (Brauneis and Goodman, 2018; Bloch-Wehba, 2021; Aoki et al., 2024; Nannini et al., 2023; Vredenburgh, 2024). However, much of this work focuses on ex post mechanisms or communication strategies and pays less attention to how explainability is defined and operationalized at the level of primary and secondary legislation in specific digital states.

#### 2.2.5 Platform governance and environmental externalities

A fifth strand treats AI-related risks as inseparable from platform governance and the environmental footprint of data-intensive infrastructures. Studies on digital platforms as new network industries show how state-sponsored platforms and common data spaces can create new forms of market power, lock-in, and state-aid tensions if access rules, interoperability, and competition safeguards are weak (Montero and Finger, 2021; Krarup and Horst, 2023; Prug and Bilić, 2024). In parallel, research on the environmental impact of AI emphasizes the energy consumption and carbon footprint of large-scale data centers and AI servers, pointing to the need to integrate climate and resource considerations into AI governance frameworks (de Vries, 2023; International Energy Agency, 2025a, 2025b; Zewe, 2025; Xiao et al., 2025). For CIS digital states, however, platform governance and environmental externalities of AI infrastructures remain largely absent from both doctrinal legal analyses and empirical research, even though national AI platforms and sovereign models are being actively promoted as pillars of digital statehood.

Taken together, these five strands establish the general conceptual coordinates of the article. The remaining question is what the existing CIS and Kazakhstan-focused literature still leaves underexplored at the intersection of legal design, data infrastructures, and administrative practice. This question is addressed in the following subsection.

### 2.3 Kazakhstan/CIS research gap and article contribution

Despite this growing body of research, three interrelated gaps remain. First, socio-technical “dark side” and AI governance frameworks have not yet been systematically applied to the emerging AI architectures of CIS digital states, where concentrated political power, centralized data infrastructures, and national AI platforms may shape risks in distinctive ways (Valle-Cruz et al., 2023; Wirtz et al., 2020, 2022). Second, while the CIS and Kazakhstan-focused literature reviewed above provides important doctrinal, transparency-related, and sector-specific insights, it remains analytically segmented and therefore does not reconstruct how design-level choices in AI legislation, integrated data infrastructures, and public-sector platforms jointly institutionalize risks ex ante across multiple domains (Konusova, 2023; Aubakirova and Moldabekov, 2024; Kabytov and Nazarov, 2025; Atabekov, 2023). Third, existing studies rarely combine normative-institutional analysis with qualitative evidence on how public officials themselves perceive digital risks and how these perceptions condition the reception of AI in public administration (Blinova et al., 2021; Stupin, 2024; Buklemishev, 2022; Bezvikonnaya, 2025; Nuruly et al., 2025).

The present article addresses these gaps by applying a socio-technical perspective to Kazakhstan as a strategically revealing CIS case and by linking analysis of AI law and national platforms with evidence on data infrastructures, organizational dynamics, explainability regimes, platform governance, environmental externalities, and civil servants’ risk perceptions.

## 3 Methodology

### 3.1 Research design and analytical scheme

The study employs a qualitative, socio-technical single-case design focused on Kazakhstan’s public sector. Single-case research is appropriate when the aim is to examine how complex institutional arrangements operate in context and when the boundaries between a policy regime and its implementation environment are not sharply demarcated (Yin, 2018). Kazakhstan is treated as a strategically revealing case because it combines accelerated legal codification of AI with a highly centralized digital infrastructure and an administratively centralizing model of digital governance (Flyvbjerg, 2006). The purpose of the design is analytical rather than statistical generalization: the case is used to refine the concept of ex ante institutionalized AI risks in digitally centralizing states and to formulate propositions for future comparative research.

The study combines a normative-institutional analysis of legal and policy documents with a secondary qualitative analysis of 53 semi-structured interviews with current and former civil servants. The object of analysis is the use of AI in Kazakhstan’s public sector as an element of a broader configuration of the digital state. The subject of the study is the set of potential negative effects arising from the interaction of: (i) special legislative regulation, including the Law of the Republic of Kazakhstan “On Artificial Intelligence” and related acts; (ii) institutional architecture, understood as the distribution of powers and accountability across state and quasi-state actors, including the Ministry; and (iii) national data and AI infrastructures, including the National AI Platform and integrated data-governance solutions such as Smart Data Ukimet. The initial theoretical framework is provided by scholarship on the “dark side” of artificial intelligence in public administration and by socio-technical approaches that emphasize the priority of political-legal, institutional, infrastructural, and organizational factors over the isolated technical characteristics of particular models (Valle-Cruz et al., 2023; Wirtz et al., 2020, 2022).

Across both empirical modules, findings are organized into five analytical blocks of hidden risks: (1) political-legal and institutional effects; (2) data and technologies; (3) organizational and managerial effects; (4) explainability/opacity; and (5) market and environmental effects. This five-part structure serves as the general analytical scheme for interpreting both regulatory documents and qualitative material. Table 1 summarizes this scheme by linking the five risk blocks to corresponding socio-technical categories in Valle-Cruz et al. (2023) and Wirtz et al. (2020, 2022), Kazakhstan-specific regulatory and infrastructural nodes, and the expected types of negative effects. Unlike the integrative schemes proposed by Valle-Cruz et al. (2023) and Wirtz et al. (2020, 2022), in which market and infrastructural aspects are partly absorbed into other categories, the present map distinguishes market and environmental effects as an independent block. It also treats national AI platforms, sovereign large language models, and AI agents as specific institutional-technological nodes of the contemporary CIS digital state.

**Table 1:** Map of hidden AI risks in Kazakhstan’s public sector.

Hidden risk block	Corresponding socio-technical categories in Valle-Cruz et al. (2023) and Wirtz et al. (2020, 2022)	Kazakhstan-specific regulatory and infrastructural nodes	Expected types of negative effects
Political-legal and institutional effects	Political, legal, and institutional risks; power shifts; accountability deficits	Law of the Republic of Kazakhstan “On Artificial Intelligence” (legal status of AI; prohibited practices; reservations and exceptions); the Ministry; concentration of regulatory, operational, and promotional roles	Depoliticization of algorithmic power; expanded regulatory discretion; weakened external oversight; conflicts of objectives arising from the combination of regulatory, operational, and promotional roles
Data and technologies	Data and technology risks; data governance; infrastructural dependence	Smart Data Ukimet; National AI Platform; Digital Family Card; integrated state information systems; sovereign large language models	Systemic bias in centralized datasets; infrastructural fragility; algorithmic stratification of households; epistemic concentration; scalability of technical failures
Organizational and managerial effects	Organizational and managerial risks	Deployment of AI agents (eGov AI, eOtinish AI, Tax Helper, QQazaq Law, etc.); KPI-based governance regime; mass AI training programs	Deskilling; displacement of expertise toward interfaces; fragmented accountability; dependence on platforms and metrics; heightened workload and burnout risks
Explainability /opacity	Transparency and explainability deficits; opacity; complexity	Explainability principles and labeling requirements in the AI Law; absence of detailed procedures for algorithmic audit, explanation, and contestation	Reduction of explainability to formal labeling; persistence of the black-box effect; transfer of interpretive and contestation burdens to the citizen
Market andenvironmental effects	Platform governance, market-structure risks, and environmental externalities (treated here as a distinct block in the present study)	National AI Platform as a centralized operator of data libraries and computing resources; absence of environmental requirements in AI regulation	Market concentration and lock-in; barriers for independent developers and researchers; externalization of climate, energy, and resource costs of AI infrastructure

The fifth block extends rather than simply reproduces the source typologies: market and environmental effects are analytically distinguished here because centralized AI platforms and national computing infrastructures make these risks especially salient in the Kazakhstani case.

### 3.2 Normative-institutional analysis of legal and policy documents

In this article, normative-institutional analysis refers to a form of doctrinal legal research that reconstructs the content of binding norms and policy instruments and examines how these norms allocate powers, duties, and accountability within an institutional architecture. Doctrinal legal research is concerned with interpreting and systematizing legal sources as they stand, while institutional analysis foregrounds how formal mandates, organizational structures, and enforcement capacities shape governance outcomes (Hutchinson and Duncan, 2012; March and Olsen, 1984). In the context of AI, this approach is suitable for analyzing risk-based regulatory logics, prohibited practices, and the distribution of responsibilities among developers, owners, users, regulators, and operators.

The normative-institutional module draws on primary legal and policy documents governing AI and digital public administration in Kazakhstan. The corpus includes the Law of the Republic of Kazakhstan “On Artificial Intelligence”; the Concept for the Development of Artificial Intelligence for 2024–2029; the Presidential Decree establishing the Ministry; implementing and programmatic documents relating to the National AI Platform; and official descriptions of Smart Data Ukimet, the Digital Family Card, sovereign LLMs, and AI agents.

In this module, the unit of analysis is the legal provision, policy instrument, or infrastructural arrangement relevant to AI status, risk classification, prohibited practices, allocation of roles, data governance, explainability, and oversight. The analysis was carried out in three steps. First, the document corpus was read to extract provisions relating to: (a) the legal status and risk classification of AI systems; (b) prohibited or restricted practices; (c) the duties and liabilities of owners, holders, users, developers, operators, and the authorized body; and (d) rules concerning data governance, explainability, labeling, human oversight, and avenues of contestation. Second, these extracted elements were mapped onto the institutional architecture of Kazakhstan’s digital state, including the Ministry, the National AI Platform, Smart Data Ukimet, the Digital Family Card, and the main quasi-state operators. Third, the results were grouped into the five analytical blocks of hidden risks used throughout the article.

The purpose of this module is reconstructive rather than evaluative: it seeks to identify how legal and institutional design pre-configures risk trajectories ex ante, rather than to assess the doctrinal coherence of the legal framework in the abstract.

### 3.3 Interview corpus and secondary qualitative analysis

The qualitative module uses a corpus of 53 semi-structured interviews with current and former civil servants of Kazakhstan, originally collected within the broader research program BR27195163 on the transformation of organizational culture, ethics, and digitalization in the civil service. Related outputs from the same program have already addressed ethical regulation and organizational culture in Kazakhstan’s civil service (Buribayev and Khamzina, 2025; Khamzina et al., 2025). The present article differs from those studies in that it re-codes a defined subset of the interview material specifically through hidden AI-risk categories. All numerical references to the qualitative corpus in this article relate only to these 53 interviews retained for the present secondary analysis. The interviews were conducted in August–September 2025, prior to the adoption of the Law of the Republic of Kazakhstan “On Artificial Intelligence” and prior to the practical deployment of the National AI Platform. Accordingly, the interview material is treated not as evidence about the functioning of particular AI systems, but as a structured corpus of civil servants’ perceptions of digitalization and digital risks at a stage when AI governance was still being institutionalized.

Sampling followed a purposive maximum-variation logic designed to capture heterogeneity across hierarchical level, institutional type, sector, and region rather than statistical representativeness (Patton, 2015). The sample included managers, experienced specialists, early-career employees, and former civil servants from central government bodies, regional akimats, subordinate organizations, and quasi-state structures representing taxation, law enforcement, the social sector, education, culture, statistics, and other fields of public administration. Inclusion criteria comprised several years of work experience in public administration and/or direct participation in digital transformation processes; the majority of respondents had substantial professional experience. A concise structural profile of the 53-interview analytic subset is provided in Table A1.

Interviews were conducted on an individual and confidential basis using a unified semi-structured guide covering the state of the civil service, ethical dilemmas, organizational culture, digital transformation, its consequences, accountability, and the risks of rapid digitalization. Participation was voluntary and based on informed consent. All interviews were transcribed verbatim, de-personalized, and processed under the same ethical protocol within research program BR27195163. In the qualitative module, the unit of analysis is the interview segment expressing a perception of digitalization, accountability, organizational pressure, or risk relevant to AI institutionalization.

The secondary analysis followed a two-cycle coding strategy (Braun and Clarke, 2006). In the first cycle, full transcripts were coded inductively for recurring motifs related to digitalization, control, platform dependence, data quality, accountability, explainability, workload, opacity, and perceptions of risk. In the second cycle, these open codes were grouped abductively into the five hidden-risk blocks derived from the normative-institutional analysis. In this way, the legal-policy module informed category consolidation, but did not determine the initial emergence of codes from the interview material.

Within the broader research program BR27195163, respondent material on civil-service ethics, organizational culture, reform implementation, and digitalization had also been summarized in SWOT (Strengths, Weaknesses, Opportunities, Threats) and PEST (Political, Economic, Social, Technological) matrices. For the purposes of the present article, only those matrix entries traceable to the same 53-interview analytic subset were used. The SWOT summaries grouped responses into strengths, weaknesses, opportunities, and threats of the contemporary civil-service environment, whereas the PEST summaries organized them under political, economic, social, and technological conditions affecting public-service practice. In the present article, these matrices are used only as auxiliary analytical summaries that facilitate retrieval, comparison, and second-cycle consolidation of risk-related themes. They are not treated as independent datasets and do not replace coding of the underlying interview material. Where matrix entries were used, they were checked against the underlying respondent material before being re-coded into the five hidden-risk blocks. Illustrative examples of this re-coding procedure are provided in Table A2, while Table A3 reports a descriptive overview of code distribution across the 53-interview analytic subset.

Because the original interview guide and the related SWOT/PEST summaries were developed for the broader topic of civil-service ethics and digital transformation rather than AI specifically, the present analysis reconstructs wider administrative risk imaginaries into which AI solutions are subsequently embedded, rather than evaluating realized harms of specific AI systems. This reduces confirmation bias, but it also limits the specificity of claims that can be made about the operational effects of already deployed AI systems.

### 3.4 Integration strategy, use of SWOT/PEST, and limitations

At the final stage, the results of the normative-institutional analysis and the secondary qualitative analysis were compared within a unified five-block scheme of hidden risks. For each block, two dimensions were examined: (1) elements of legal regulation, institutional design, and data architecture that generate corresponding risks ex ante; and (2) patterns of risk perception reconstructed from the interview corpus. Convergences between the two modules were interpreted as indicators of how strongly particular risks are institutionalized, whereas divergences were treated as analytically important gaps between formal design and administrative perception. On this basis, the final map of hidden AI risks in Kazakhstan’s public sector was constructed.

The qualitative character of the study and its focus on a single national context are deliberately maintained. The design does not seek statistical representativeness or causal evaluation of operational AI performance; rather, it supports an ex ante mapping of institutionalized risk trajectories and risk imaginaries in a rapidly centralizing digital state. Because the original SWOT/PEST matrices were developed for the broader topic of civil-service ethics and digital transformation rather than AI specifically, they are used in this article only as auxiliary contextual materials and not as independent evidence of the functioning or effects of particular AI systems. In addition, all numerical references to the qualitative material in the present article relate only to the 53 interviews retained for the secondary analysis. This methodological stance corresponds to the purpose of the article, namely, to demonstrate how negative effects of AI in Kazakhstan’s public sector may be entrenched within a preconfigured regulatory architecture and established practices of digital governance. In the Results section, interview evidence is presented in a structured form through the same five hidden-risk blocks used in the documentary analysis, with descriptive indications of thematic distribution and differentiated respondent-type illustrations.

## 4 Results

This section presents the empirical findings in two steps. Section 4.1 reconstructs the legal and institutional architecture through which AI-related risks are configured in Kazakhstan’s public sector. Section 4.2 presents interview-based evidence on how civil servants perceive digitalization, control, accountability, infrastructural dependence, and opacity in ways directly relevant to AI institutionalization. The analysis remains primarily ex ante: given the recency of Kazakhstan’s AI Law and the staged deployment of the National AI Platform, the findings are interpreted as design-level risk trajectories rather than as a full evaluation of realized harms.

### 4.1 Legal and institutional framework

The legal-institutional findings should be read as an analysis of implementation architecture. Kazakhstan’s AI Law adopts a risk-based logic broadly consonant with the EU AI Act and is formally aimed at mitigating harmful uses of AI. At the same time, several key safeguards, particularly detailed procedures for audit, contestation, independent oversight, and operational secondary regulation, remain weakly specified at the implementation stage. As a result, the legal framework simultaneously declares responsible AI principles and creates conditions under which latent risks may be institutionalized before large-scale harms become directly observable.

#### 4.1.1 Political-legal and institutional effects

Three documentary findings are most relevant here: the legal instrumentalization of AI, the open-ended structure of key prohibitions, and the concentration of regulatory and infrastructural roles within the Ministry. Together, these features define the political-legal and institutional risk profile of Kazakhstan’s emerging AI regime.

The instrumental qualification of AI as an “object” and a “tool,” combined with its simultaneous integration into processes of allocating rights and resources, shifts attention away from the political and legal nature of decisions toward their technical shell. This generates a neutrality effect: decisions mediated by technical systems may be perceived as more objective and less contestable even when they reproduce existing inequalities, bureaucratic asymmetries, or selective patterns of state visibility. In such a configuration, the technical mediation of a decision does not reduce its public significance, but it may conceal the fact that the decision remains politically consequential and normatively loaded.

The open-ended construction of prohibitions and the possibility of their adjustment through special legal regimes reinforce regulatory discretion. The institutional concentration of the roles of regulator, infrastructure operator, and AI promoter within a single agency creates a persistent conflict of objectives. Within such a design, the prioritization of accelerated technological deployment over the construction of robust accountability mechanisms appears not incidental, but structurally embedded. External oversight by parliament, the judiciary, or independent regulators for data protection and human rights is weakened because a significant share of information, expertise, and infrastructure is concentrated within a single executive center.

These documentary findings indicate that political-legal risks are rooted not only in possible implementation errors but in the legal definition of AI, the open-ended framing of key prohibitions, and the concentration of roles around digital-state infrastructures. The current regime therefore does not merely regulate risk; it also pre-configures the conditions under which certain risks may become normalized in public administration.

#### 4.1.2 Data and technologies: centralization, fragility, stratification

Documentary analysis identified three interconnected technological risks: centralized data dependence, algorithmic stratification through proactive scoring, and epistemic concentration through sovereign-model integration. These risks arise from the interaction of Smart Data Ukimet, the Digital Family Card, the National AI Platform, and sovereign large language models being integrated into public services.

The Digital Family Card is illustrative in this respect. In official discourse, it is presented as an instrument for implementing the principle that no one should be left behind: the system identifies households in difficult circumstances, informs them about support measures, and may simplify access to services. Yet it is precisely this seemingly benevolent function that contains latent threats. Multidimensional household scoring based on hundreds of indicators is inevitably associated with classification errors. A household that does not cross a formal threshold on one parameter may nevertheless remain socially vulnerable due to informal employment, indebtedness, chronic illness, or unstable family circumstances. Under conditions of procedural automation, such false negatives become difficult to detect from the citizen’s side: the absence of a notification or service is experienced as a natural and non-contestable outcome.

The operating logic of the Digital Family Card also presupposes the creation of an extremely detailed digital profile of the household, including sensitive information on income, employment, family composition, housing conditions, and health-related variables. Initially, such a profile is justified by the purposes of social support; however, technologically nothing prevents its secondary use for other purposes. These could include assessing creditworthiness, targeting control and inspection activities, segmenting citizens by degrees of reliability or risk, or enabling personalized communication and behavioral targeting. In the absence of clearly defined legal restrictions on cross-contextual data use and transparent rules governing access by different agencies, there emerges a risk of durable algorithmic stratification. Households may become fixed in certain data-defined classes, and this infrastructural status may shape not only access to social services but the broader modality of interaction with the state.

This danger is illustrated by the reported exclusion of approximately 90,000 incorrect records from housing waiting lists (Madiyev, 2025). On the one hand, analytical tools do make it possible to identify anomalies and abuses. On the other hand, the criteria of “incorrectness” and “abuse” are themselves embedded in classificatory models that are not publicly explained and are not subject to independent external audit. For the state, such tools appear as instruments for governing fairness; for citizens, they remain opaque and difficult to contest.

A further layer of risk arises from sovereign large language models integrated into public services, AI agents, and potentially into lawmaking support and legal advisory functions. In Kazakhstan, this sovereign-model strategy is not confined to the state language alone: official descriptions of KazLLM present it as a multilingual model for Kazakh, Russian, and English, with additional Turkish support, while current policy discourse on sovereign AI deployment refers more broadly to public-sector LLM services operating in Kazakh and Russian (ISSAI, 2024; Madiyev, 2025). Their promotion as models that “understand language and context” generates at least three interrelated effects. First, such models are likely to reproduce institutionally dominant discursive patterns concerning the state, history, national identity, development, and public priorities, because a substantial share of their training material is drawn from national websites, media, official documents, and locally curated discussions. Second, their use as universal interfaces for legal and political information may reduce source pluralism. If citizens receive explanations primarily from a single AI agent trained on nationally curated corpora and integrated with official databases, alternative interpretations, critical commentary, and minority positions may become comparatively marginalized. Third, even without direct hard filtering, the selection of training corpora and the prioritization of official sources may produce a soft but persistent privileging of what the model comes to treat as reliable and typical knowledge. In this sense, sovereignty in AI may generate not only control over infrastructure and data, but also a structurally privileged position for certain official or institutionally curated interpretations of social reality within state-facing cognitive tools. This claim is structural rather than intentional: we do not posit a centrally coordinated strategy of AI-enabled social control; rather, we argue that epistemic steering and asymmetrical visibility may emerge from centralized curation of data, interfaces, and model governance even in the absence of explicit coercive intent.

Taken together, the data and technologies block generates not only classical risks of bias and technical error, but also a broader configuration of algorithmically mediated stratification and norm-setting. Centralized data infrastructure, proactive services based on big data, and sovereign LLMs embedded in the public sector form a stable circuit in which the structural properties of data and institutionally dominant discourses may be translated into managerial decisions and law-enforcement practice. These effects remain largely latent because the official agenda emphasizes efficiency, inclusiveness, and sovereignty, whereas issues of systemic bias, digital stratification, and epistemic concentration remain weakly articulated in regulation and public debate.

#### 4.1.3 Organizational and managerial effects: deskilling, dependence, shifts in accountability

The documentary material indicates three organizational effects: interface-centred expertise, fragmented responsibility, and metricized performance control. These effects are visible across existing and planned public-sector AI services, including eGov AI assistants, eOtinish AI, Tax Helper, QQazaq Law, AI Therapist, and related tools.

One of the key latent effects of this configuration is a shift in the locus of expertise. AI agents and assistants are designed to provide ready-made or near-ready solutions: Tax Helper generates tax prompts and interpretations of norms; eGov AI assists in selecting services and formulating requests; legal assistants generate template responses and drafts; and agency-specific tools support the analysis of large datasets and citizen appeals. Within such an architecture, the key skill increasingly becomes the ability to formulate prompts correctly, interpret outputs, and align them with bureaucratic procedures, rather than the accumulation of deep sector-specific legal, social, or administrative expertise. The risk of deskilling is therefore cumulative. Younger cohorts entering the civil service under conditions of pervasive AI assistance may acquire a reduced version of the professional role in which independent reasoning, contextual judgment, and the motivation to develop substantive knowledge are gradually weakened.

A second dimension concerns fragmented accountability. At least three groups of actors are involved in AI-mediated public decisions: the developer or operator, often a quasi-state IT company or consortium; the user agency, which relies on the AI system in routine practice; and the citizen, whose rights and interests are affected by the output. Formally, the law differentiates duties and liabilities among owners, holders, users, and the authorized body. In practice, however, the absence of detailed secondary regulation governing data and algorithm audit, mandatory logging of model versions and parameters, and clear rules for human intervention creates a zone of managerial uncertainty. In the event of an adverse outcome, such as an erroneous refusal of a service, exclusion from a waiting list, or a discriminatory scoring result, a situation arises in which responsibility is effectively without an addressee. Developers refer to technical compliance with specifications; user agencies invoke the objectivity of the algorithm and their lack of capacity to assess its internal logic; citizens remain unable to reconstruct the chain through which the decision was produced.

A third dimension concerns the use of AI tools for monitoring and evaluating the performance of public authorities and individual civil servants. Already at present, Smart Data Ukimet and related analytical cases are used to assess the effectiveness of anti-poverty measures, the quality of akimats’ performance, the fairness of housing allocation, and other policy areas. The eOtinish platform enables the analysis of citizens’ appeals by region and category and identifies bottlenecks in the work of agencies. The next logical step is the more detailed quantification of performance through AI-based indicators, including the processing time of appeals, the share of automated decisions, predicted corruption risks, and the identification of deviant employee behavior. In this configuration, AI begins to function not only as a service-delivery tool but as a norm-setting mechanism for professional behavior. Civil servants are incentivized to optimize what is algorithmically visible and measurable, even when such optimization undermines substantive responsiveness to complex citizen needs.

Taken together, the organizational and managerial risks arising at the intersection of AI agents, training programs, and the new legal architecture complement the political-legal and technological dimensions of AI risks. The shift of the locus of expertise toward algorithmic systems, the fragmentation of accountability among developers, agencies, and citizens, and the normalization of professional behavior through AI-generated metrics create a regime of hidden dependence of the public apparatus on specific configurations of AI systems. Within such a regime, technological optimism and the course toward extensive AI integration risk becoming institutionalized as an organizational dogma, while issues of preserving professional autonomy, accountability, and substantive public-service quality recede into the background.

#### 4.1.4 Explainability/opacity

The documentary findings in this block concern a tension between formal transparency requirements and substantively weak explainability. The combination of labeling duties, general principles of transparency, and under-specified procedures for audit and contestation creates a configuration in which explanation may remain largely symbolic.

The core problem concerns the meaning of explainability. The labeling envisaged by the law is effectively a binary indicator: an output is either created wholly or partially using AI, or it is not. Yet this does not disclose what role AI actually played in the process. It remains unclear whether AI merely assisted in editing or translation, or whether it shaped the substantive outcome of a decision by prioritizing applications, scoring a household, or selecting a service recipient. Substantive explainability would require, at minimum, meaningful information about the purpose of the model, the main data sources and assumptions underlying its outputs, and the reasons why a particular result was produced in a particular case. In the current legal design, the first element is partially recognized through general principles, whereas the second and third are left to future technical and secondary-regulatory choices.

In this configuration, the labeling requirement may perform a largely symbolic function. The presence of a marker such as “created using AI” may be perceived as sufficient compliance with transparency requirements even though the crucial questions remain unanswered: how exactly did the algorithm influence the outcome, which factors were taken into account, and whether alternative decisions would have been possible on the basis of the same input data? This means that the system may become formally transparent while remaining non-explainable in any legally or socially meaningful sense. Moreover, the burden of cognition is shifted toward the citizen: the individual is informed that AI was used, but is not equipped with the information or procedural tools necessary to evaluate or challenge the decision.

#### 4.1.5 Market and environmental effects

The institutionalization of the National AI Platform as a unified infrastructure creates a distinct cluster of latent risks situated at the intersection of market structure and environmental externalities.

At the core of this cluster is the role of the National AI Platform as a centralized market and infrastructural node. The platform is intended to provide a controlled environment for the collection, processing, storage, and dissemination of data libraries, as well as for the development, training, and pilot deployment of AI models. State bodies supply data libraries and provide the platform operator with access to relevant datasets; the operator processes these datasets and offers AI-related services on the basis of the national platform. At the level of stated goals, this model aims to ensure technological sovereignty, standardization, and lower barriers to development. In practice, however, it creates preconditions for a de facto monopolistic gateway to government data and computing resources. The law does not guarantee equality of conditions for access, tariff neutrality, or the absence of preferential treatment for affiliated structures. Because the platform operator is appointed by the government and has the status of a quasi-state organization, asymmetries between the operator and private or academic initiatives are objectively reinforced.

A related risk is innovation lock-in. Early architectural choices regarding data formats, access protocols, and baseline models may become entrenched as de facto standards and subsequently constrain the adoption of alternative or more advanced technological solutions. If state bodies and quasi-state actors are encouraged, whether formally or informally, to use the National AI Platform as the approved environment for training and deployment, the space for alternative cloud providers, federated solutions, or independent data architectures is correspondingly narrowed.

The environmental dimension of this configuration remains almost entirely outside the scope of Kazakhstani AI regulation. Large-scale AI computation is increasingly associated internationally with growing data-center energy consumption, greenhouse-gas emissions, and water use for cooling systems. In Kazakhstan, the expansion of computing capacity required by large data platforms and AI infrastructures will inevitably increase cumulative pressure on national energy resources. Unlike the EU’s emerging regulatory approach, which is moving toward the integration of sustainability and resource-efficiency considerations into AI governance, Kazakhstani regulation currently lacks provisions that would incorporate the environmental footprint of AI into a risk-oriented oversight framework.

Taken together, the institutional, market, and environmental parameters of the National AI Platform form a unified latent-risk configuration that is weakly articulated in the current regulatory regime. At the level of discourse and legal principles, the emphasis is placed on technological modernization, standardization, and sovereign capacity. At the level of implementation architecture, however, durable barriers to competition may emerge around the platform’s exclusive status, while the environmental costs of centralized AI infrastructure may remain external to AI governance altogether.

### 4.2 Interview findings

The interview findings do not document the operational performance of particular AI systems. Rather, they reconstruct how civil servants at different levels perceive digitalization, control, accountability, infrastructural dependence, and opacity, that is, the administrative environment into which AI tools are now being introduced.

To provide a more structured presentation of the qualitative evidence, the interview findings are reported below according to the same five hidden-risk blocks used in the documentary analysis. In addition to illustrative quotations, the analysis indicates the descriptive distribution of each block within the 53-interview analytic subset. These descriptive indications are not intended as claims of statistical representativeness. Rather, they show how widely a given motif appeared across the coded corpus and how its articulation varied across respondent groups. A descriptive overview of second-cycle code distribution across the 53-interview analytic subset is provided in Table A3.

To preserve anonymity while retaining analytical differentiation, the evidence is presented through respondent-type categories such as central-government managers, regional specialists, quasi-state actors, and former civil servants. The quotations reproduced below are translated and de-identified respondent excerpts from the interview corpus. They are presented as illustrative expressions of recurrent motifs across respondent categories rather than as exhaustive statements.

#### 4.2.1 Political-legal and institutional effects in interview perceptions

The political-legal and institutional block appears in the interview corpus through recurring references to hierarchy, informal influence, the need for clearer rules, and weak trust in substantive rather than merely declarative accountability. These themes were especially visible in managerial and mid-career narratives. Managers and more experienced respondents more often articulated digital transformation in terms of institutional design and implementation capacity, emphasizing both the opportunities of reform and the need for clearer procedural frameworks and stronger resource support. As one respondent put it: “Reforms create opportunities, but resources are scarce; clearer rules are needed.” [Male manager].

At lower and middle levels of the bureaucracy, the same block appeared less as an abstract regulatory problem than as an everyday experience of hierarchy, limited flexibility, and dependence on informal channels of influence. In such accounts, digitalization was not described primarily as a neutral modernization process, but as an additional layer imposed on already existing asymmetries of authority. A recurrent perception was that formal rules alone do not exhaust how decisions are made and that initiative remains constrained by vertical structures: “Without connections it is difficult to move initiatives forward; hierarchy is too strong and there is no flexibility.” [Manager over 46].

The interview corpus also indicates weak trust in substantive accountability and low confidence in the safety of internal contestation. This is analytically important because it suggests that AI systems are entering an environment in which responsibility is already difficult to articulate and challenge in non-formal terms. As one respondent noted, “It is not always safe to speak about violations; the collective often exerts pressure.” [Mid-career employee] These interview-based perceptions do not yet constitute evidence of AI-specific legal harms in operation. However, they show that many of the institutional preconditions of such harms—hierarchical asymmetry, low contestability, and weak trust in substantive accountability—were already present before the large-scale rollout of the current AI agenda.

#### 4.2.2 Data and technologies in interview perceptions

The data-and-technologies block is reflected in the interview corpus through perceptions of dependence on integrated systems, infrastructural fragility, platform overload, and anxiety concerning data security. These motifs were especially visible in comments by managers and younger employees, who described digital infrastructures as simultaneously necessary for administrative work and vulnerable to disruption, uneven capacity, and misuse. In this respect, the interview material complements the documentary analysis by showing how centralized infrastructures are experienced from within the bureaucracy rather than only designed from above.

A recurrent theme was dependence on integrated systems whose failures or limitations could slow work, distort routines, or intensify uncertainty. Respondents did not describe these issues in the vocabulary of AI infrastructure as such, since most interviews predated the practical rollout of key AI initiatives, but they did repeatedly point to system overload, uneven preparedness, and the difficulty of working within layered digital environments. Such accounts are directly relevant to the AI agenda because they indicate that new AI services will be embedded in institutions that already experience digital dependence as a practical vulnerability rather than as a purely technical matter.

Concerns about data handling and infrastructural insecurity were also salient. In some responses, technological dependence was articulated together with distrust in organizational practices and fears of improper data use or leakage. As one younger respondent stated, “Decisions are made through personal connections, and there is a real danger of data leakage.” [Young employee] Taken together, these materials show that centralized and integrated infrastructures are experienced not only as enablers of efficiency, but also as sources of dependence, fragility, and unequal exposure to system failure.

#### 4.2.3 Organizational and managerial effects in interview perceptions

The organizational and managerial block is visible in the interview corpus through recurrent references to formalism, reporting burden, intensified visibility requirements, and digitalization as an instrument of objective control. These themes were particularly salient among younger staff and were also recurrent in managerial accounts. Several respondents described digital transformation less as a process of empowerment than as an expansion of measurable obligations, procedural discipline, and pressure to generate reportable outputs.

One recurring motif was the burden of formalization: digital systems were experienced as adding procedural layers to already demanding administrative routines. For younger employees in particular, digitalization was associated with friction rather than simplification. As one respondent observed, “There are too many formalities; they slow work down, and there is not enough information or resources to apply the law properly.” [Employee under 35] In this register, digitalization appears as an extension of bureaucratic load rather than a reduction of it.

At the same time, some respondents framed digital services as increasing transparency while also intensifying organizational pressure and slowing work. This ambivalence is important for understanding how AI may later be received in the public sector: technologies are valued for their capacity to make behavior visible and controllable, yet they are also experienced as creating additional friction and dependence. As one female manager noted, “Digital services make work more transparent, but sometimes they also slow it down.” [Female manager] What appears here is not yet deskilling in the strict sense of routine AI substitution, but an organizational environment in which expertise is already being displaced toward procedural visibility, measurable outputs, and technically mediated control.

#### 4.2.4 Explainability and opacity in interview perceptions

A separate and analytically important block concerns explainability and opacity. Across respondent groups, digital systems were often experienced as authoritative yet only partially intelligible. Even where respondents did not explicitly use the language of “explainability” or “black boxes,” their descriptions repeatedly pointed to systems whose outputs had to be accepted, followed, or worked around without a clear understanding of how a result had been produced or why a particular outcome had occurred.

A notable finding across respondent groups is that digital systems are often experienced as authoritative yet only partially intelligible. This is analytically important because it indicates that opacity is not introduced by AI alone; rather, AI is likely to enter an administrative environment in which partial intelligibility, deference to system outputs, and limited contestation are already normalized.

The opacity block therefore appears in the qualitative material not primarily as a demand for sophisticated technical disclosure, but as a practical condition of everyday administrative work. Systems may enhance visibility from above while remaining only partly intelligible to those who operate them and to those affected by their outputs. This indicates that future AI-related explainability problems in Kazakhstan are likely to be shaped not only by the complexity of models, but also by pre-existing institutional habits of limited interpretability and constrained contestation.

#### 4.2.5 Market and environmental effects in interview perceptions

Compared with the other four hidden-risk blocks, market-structural and environmental concerns were only weakly articulated in the interview corpus. Respondents more readily described dependence on centralized systems, uneven technical capacity, and infrastructural fragility than competition-related or sustainability-related issues as such. This relative absence is analytically meaningful: it suggests that broader market concentration and environmental externalities of public-sector AI infrastructures remain weakly problematized within administrative perceptions, even though they are visible at the level of institutional design.

Where this block did surface indirectly, it did so mainly through references to dependence on centralized infrastructures and the unequal distribution of technical resources across organizations. These perceptions do not amount to an explicit critique of market lock-in or environmental costs. However, they help explain why such issues may remain under-articulated in administrative discourse even as they become increasingly important at the level of institutional design and national platform governance.

Table 2 provides a structured overview of how interview-based themes are distributed across the five hidden-risk blocks and which motifs were most visible across respondent groups.

**Table 2:** Descriptive distribution of interview-based themes across the five hidden-risk blocks.

Hidden-risk block	Main recurring interview themes	Descriptive distribution within the 53-interview analytic subset	Most visible respondent groups	Illustrative excerpt or motif
Political-legal and institutional effects	Hierarchy, informal influence, need for clearer rules, weak trust in substantive accountability, and low safety of internal contestation	Present across multiple managerial and mid-career interviews, especially in comments on hierarchy, informal influence, limited flexibility, and weak contestability	Managers; mid-career employees	“Reforms create opportunities, but resources are scarce; clearer rules are needed.” [Male manager]
Data and technologies	Dependence on integrated systems, platform overload, data-leakage anxiety, and infrastructural fragility	Present across managerial and younger-employee interviews, especially in comments on system dependence, overload, uneven preparedness, and data insecurity	Managers; younger employees	“Decisions are made through personal connections, and there is a real danger of data leakage.” [Young employee]
Organizational and managerial effects	Formalism, reporting burden, pressure for visible outputs, and digitalization as an instrument of objective control	Especially concentrated in staff interviews, with repeated confirmation in managerial accounts concerning formalism, reporting burden, and measurable-output pressure	Employees under 35; managers	“There are too many formalities; they slow work down, and there is not enough information or resources to apply the law properly.” [Employee under 35]
Explainability/opacity	Partial intelligibility of systems, black-box experience, normalized opacity, and limited everyday contestation	Present across respondent groups, typically expressed as partial intelligibility and black-box experience rather than in the technical vocabulary of explainability	Central-government managers; regional specialists	Recurring descriptions of digital systems as authoritative yet only partly intelligible in everyday administrative work
Market and environmental effects	Centralized dependence, uneven technical capacity, and indirect awareness of infrastructural concentration	Rarely articulated explicitly as market or environmental concerns; inferable mainly from comments on centralized dependence, unequal technical capacity, and infrastructural concentration	Senior managers; quasi-state actors	Indirect references to dependence on centralized infrastructures and unequal organizational access to technical capacity

Descriptive distribution labels refer to the spread of a motif across the 53-interview analytic subset and are used in place of formal frequency statistics. They synthesize recurring patterns identified through transcript coding and second-cycle consolidation into the five hidden-risk blocks.

Taken together, the documentary and interview evidence point in the same direction: risks are most visible where centralized infrastructures, weakly operationalized safeguards, and established administrative routines intersect. At the same time, the interview corpus shows that many civil servants articulate these vulnerabilities in narrower operational terms than the broader socio-technical and legal analysis would suggest. This gap becomes the starting point for the discussion that follows.

## 5 Discussion and policy implications

### 5.1 Interpretation of findings in relation to prior research

Where Section 4 reported the documentary and interview-based findings in a structured empirical form, the present subsection interprets their broader significance for scholarship on AI governance, digital bureaucracy, and institutionalized risk. The discussion therefore moves from case-specific evidence to conceptual implications rather than restating the findings as such.

The most important pattern revealed by the case is the convergence between the documentary and qualitative modules in four respects. First, both point to fragmented accountability: the legal architecture differentiates roles without fully operationalizing audit, explanation, and contestation, while respondents describe environments in which responsibility is already difficult to locate and challenge. Second, both indicate growing infrastructural dependence: policy design centralizes data and platform functions, while civil servants describe dependence on systems, overload, and organizational fragility. Third, both modules point to opacity: formal transparency risks being reduced to symbolic labeling, while respondents already experience digital systems as authoritative yet only partly intelligible. Fourth, both indicate the normalization of digital control: legal and infrastructural arrangements expand the capacity to classify, score, monitor, and rank, while interviewees describe managerial environments increasingly oriented toward visible, measurable, and standardized conduct.

The divergence between the two modules is equally important. The legal framework presents AI primarily through principles, classifications, and modernization goals, whereas the interview material shows that many civil servants still perceive digital risks through narrower operational lenses such as cyber threats, system overload, technical failures, and reporting pressure. This gap helps explain why latent AI risks may remain weakly articulated even as they become institutionally entrenched. In this sense, the Kazakhstani case shows not only how risks are built into legal and infrastructural design, but also how they may remain under-problematized within everyday administrative practice.

The argument should therefore be read as one about emergent structural effects of legal design, infrastructural centralization, and administrative routines rather than about a centrally coordinated strategy of AI-enabled social control.

This interpretation supports a socio-technical reading in which political-legal design, centralized infrastructures, and administrative culture matter at least as much as the technical properties of models. It therefore resonates with international literature on the dark side of AI in public administration, which has consistently shown that the most consequential harms stem from the institutional and infrastructural contexts in which algorithms are embedded rather than from isolated technical failures alone (Valle-Cruz et al., 2023; Wirtz et al., 2020, 2022; Busuioc, 2021). The Kazakhstani case extends this line of argument by showing how a formally risk-based legal regime, broadly consonant with the EU AI Act, may nonetheless coexist with concentrated institutional roles, under-specified procedural safeguards, and centralized platform architectures.

More specifically, the findings extend at least four strands of prior research. First, they support work on data infrastructures and platform governance by showing that risks are not exhausted by model bias but arise from centralized data architectures, proactive scoring systems, and platform-based control over access to compute and datasets (Ducuing, 2020; Montero and Finger, 2021; Krarup and Horst, 2023). Second, they reinforce organizational analyses of digital bureaucracy by demonstrating that AI enters administrative settings already shaped by hierarchy, metricization, and audit-oriented performance cultures, thereby shifting expertise toward interfaces and fragmenting accountability (Blinova et al., 2021; Buklemishev, 2022; Stupin, 2024). Third, the findings refine the explainability literature by showing that, in this case, opacity is likely to be institutionalized not only through complex models but also through legal designs that reduce explainability to labeling and leave contestation weakly operationalized (Kabytov and Nazarov, 2025; Aoki et al., 2024; Vredenburgh, 2024). Fourth, they extend the still limited discussion of market and environmental externalities of public-sector AI by connecting national platform governance to lock-in effects and uninternalized infrastructure costs (de Vries, 2023; International Energy Agency, 2025a, 2025b; Xiao et al., 2025).

In this article, this configuration is described as regulatory techno-optimism. The term refers to a mode of public policy in which the authority responsible for regulation simultaneously positions itself as a driver of technological modernization and national competitiveness. In that sense, the concept intersects with critiques of technological solutionism and innovation-friendly governance, as well as with studies of indicator-based public management in which formal performance metrics displace substantive accountability (Morozov, 2013; Bevan and Hood, 2006; Dahler-Larsen, 2014; OECD, 2025; OECD and UNESCO, 2024).

The Kazakhstani case shows that regulatory techno-optimism is not merely a matter of official discourse. It is reproduced institutionally through the concentration of the roles of regulator, operator, and technology promoter; infrastructurally through the centralization of data and compute; and organizationally through administrative environments that already normalize hierarchy, metricization, and limited contestability. The asymmetry of risk perceptions reconstructed from the interviews reinforces this point: upper levels of the bureaucracy more often frame digitalization as an instrument of governability, while lower levels more often experience it as an additional layer of formalism, overload, and dependence.

Taken together, these findings suggest that in digitally centralizing post-Soviet states the decisive question is not whether AI is formally regulated, but how regulatory principles are translated into infrastructures, operating routines, and opportunities for explanation, appeal, and independent oversight. Kazakhstan is analytically significant not simply because it has adopted a dedicated AI law, but because it combines a formally risk-based legal regime with centralized data infrastructures, sovereign-model ambitions, and an administrative culture already oriented toward vertical control and metricized visibility. The case therefore speaks not only to Kazakhstan, but to a wider class of digitally centralizing states in which AI governance may be formally aligned with international standards while remaining substantively weak in contestability, infrastructural pluralism, and external oversight.

At the same time, the argument advanced here has clear scope conditions. The article does not claim that all identified risks have already materialized as measurable harms, nor that the Kazakhstani trajectory can be mechanically generalized to all post-Soviet jurisdictions. Rather, it identifies ex ante risk trajectories in a single, strategically revealing case on the basis of legal-institutional analysis and qualitative evidence on administrative risk perceptions. Because the interview material predates the large-scale rollout of key AI initiatives, the qualitative findings support interpretation of organizational and perceptual preconditions for AI governance, not causal evaluation of specific AI systems in operation.

### 5.2 Policy implications for the implementation of Kazakhstan’s AI regime

The importance of the foregoing findings is not merely diagnostic. Because the risks identified in Section 4 are institutionalized at the level of legal design, centralized infrastructures, and administrative routines, secondary legislation and oversight architecture remain the principal levers through which their trajectory can still be altered. Against this background, the identified risk map and the empirical patterns of digitalization make it possible to delineate a set of nodal points at which adjustments to secondary legislation and institutional oversight mechanisms could alter the trajectory of AI risk institutionalization. In essence, this concerns a targeted reconfiguration of the already emerging regime of regulatory techno-optimism.

- Formalization of the status of algorithmically mediated decisions and the right to appeal. It is necessary to procedurally formalize a right to algorithmic appeal in high-risk domains such as social protection, taxation, and justice. This should include an obligation for the authority to provide an intelligible explanation of the decision and to review the case without reliance on AI. Decisions made with the use of AI should be expressly recognized as a form of exercising public authority and therefore subject to the same standards of legality and accountability as conventional administrative acts.
- Independent algorithmic and data audit. Mandatory external audits should be introduced for high-risk systems, including the Digital Family Card, key eGov services, and Smart Data Ukimet analytics, with a focus on bias, robustness, and compliance with legal constraints. Secondary legislation should also regulate lifecycle logging, including model versions, datasets used, parameter settings, and human interventions, and ensure the availability of these logs to oversight bodies and courts.
- Separation of the roles of regulator, operator, and technology promoter. It appears necessary to institutionally separate the functions of rulemaking and oversight, on the one hand, and the operation of AI infrastructures, including the National AI Platform, on the other. This may be achieved through the involvement of independent bodies responsible for data protection, human rights, and competition, together with restrictions on combining regulatory and investment interests within a single authority.
- Deepening explainability beyond formal labeling. There is a need to develop standards of multi-level explainability for high-risk systems, including both system-level and case-specific explainability. The label “created using AI” should function as an entry point to substantive information about the nature of algorithmic intervention, especially in relations between the state and the citizen.
- Data governance and the prevention of algorithmic stratification. For proactive services such as the Digital Family Card and analogous systems, it is advisable to introduce human-rights impact assessment procedures, including mandatory analysis of classification errors and disproportionate effects on vulnerable groups. Strict regulation of the cross-contextual use of household digital profiles is also required, together with transparency of profile-formation mechanisms, clear access rules, and enforceable purpose limitation.
- Support for professional competence and human oversight. Training programs for civil servants should include modules on the critical use of AI and the management of algorithmic risks in order to compensate for deskilling tendencies identified in the interviews. Regulatory frameworks should entrench the principle of mandatory human involvement in decisions affecting rights and freedoms and should prohibit fully automated decision-making in such domains.
- Market and environmental parameters of the National AI Platform. Rules of non-discriminatory access to the resources of the National AI Platform should be established for private and academic actors in order to prevent the displacement of independent initiatives. AI regulation should also integrate minimum requirements for environmental reporting, including accounting for energy consumption, carbon footprint, and the energy efficiency of AI infrastructures.

The listed nodal points directly address the deficits identified in both the normative analysis and the empirical data. These include the gap between declarative accountability and actual governance practices, the weakness of procedural explainability, the orientation toward control through metrics, and the absence of external channels for influencing data and algorithmic architectures. Their institutional elaboration and entrenchment in secondary legislation are necessary if AI in Kazakhstan’s public sector is to avoid further deepening the pre-existing risks of the digital-state configuration.

These implications should therefore be read as design-oriented priorities for the next stage of implementation, rather than as a retrospective assessment of already mature AI deployment in Kazakhstan’s public sector.

## 6 Conclusion

This article has argued that the risks of AI in Kazakhstan’s public sector are structured primarily by legal design, centralized data infrastructures, and administrative routines rather than by isolated technical properties of models alone. Using a five-block map of hidden risks, the study shows that the emerging AI regime combines risk-based regulation with concentrated institutional roles and weakly operationalized safeguards for explanation, audit, and contestation.

The significance of these findings is both analytical and practical. Analytically, the article demonstrates that a formally risk-based AI regime may still institutionalize vulnerabilities when legal principles are not translated into operational safeguards and when centralized infrastructures concentrate power, data, and interpretive authority. Practically, the findings suggest that the future trajectory of AI in Kazakhstan’s public sector will depend less on additional declarations of principles than on the operationalization of safeguards in secondary regulation and oversight practice, including appealable algorithmically mediated decisions, independent audit of data and models, stronger human oversight, more substantive standards of explainability, and fairer governance of national AI infrastructures.

The study has three principal limitations. First, it is based on a qualitative design and a single national case and therefore does not claim statistical representativeness. Second, the interview material predates the large-scale rollout of key AI initiatives, which means that the article reconstructs institutionalized risk trajectories and administrative perceptions rather than realized harms of specific AI systems in operation. Third, the empirical perspective is limited to civil servants and does not yet include citizens, contractors, or vulnerable groups most directly affected by algorithmically mediated decisions. Future research should therefore combine comparative case studies, mixed-method designs, and user-centered evidence in order to test how deskilling, algorithmic stratification, opacity, and fragmented accountability evolve as AI systems move from legal design to routine administrative use. In this sense, the Kazakhstani case should be understood not only as a national case study, but also as a starting point for a broader comparative research agenda on institutionalized AI risks in public administration.

## Data Availability

The original contributions presented in the study are included in the article/Supplementary material, further inquiries can be directed to the corresponding author.
