# Supplementary material for: Potential negative effects of artificial intelligence in Kazakhstan’s public sector: an analysis of hidden risks
Source: Front Artif Intell. 2026 May 25;9:1781993. doi: 10.3389/frai.2026.1781993 (PMC13243255; doi:10.3389/frai.2026.1781993)
Supplement: Supplementary file 1 [file Data_Sheet_1.docx]

**Appendix A. Interview Corpus, Coding Overview, and Recoding Illustrations**

The appendix provides three forms of methodological support for the qualitative module. Appendix Table A1 presents a compact profile of the 53-interview analytic subset. Appendix Table A2 illustrates how interview, SWOT, and PEST materials were re-coded into the hidden-risk blocks used in the main analysis. Appendix Table A3 provides a descriptive overview of second-cycle code distribution across the same analytic subset, including the number of coded segments, the number of interviews in which each block appeared, and notes on respondent-type distribution.

**Appendix Table A1. Profile of the 53-interview analytic subset**

| **Analytical dimension** | **Coverage in the 53-interview analytic subset** | **Why it matters for interpretation** |
| --- | --- | --- |
| Employment status | Current and former civil servants | Captures both internal and retrospective perspectives on digitalization, accountability, and administrative change. |
| Institutional location | Central government bodies, regional akimats, subordinate organizations, and quasi-state entities | Ensures variation across hierarchical levels and organizational settings of public administration. |
| Position profile | Managers, experienced specialists, and early-career employees | Makes visible hierarchical differences in risk perception, reporting pressure, and exposure to digital control. |
| Sectoral spread | Taxation, law enforcement, the social sector, education, culture, statistics, and other public-service domains | Allows comparison across administrative fields exposed to different intensities of digitalization and data integration. |
| Experience profile | Respondents with several years of public-service experience, with a majority reporting substantial tenure | Supports interpretation of perceptions as institutionally informed rather than episodic or incidental. |
| Regional coverage | Multiple regions and administrative centers | Reduces the risk that the qualitative material reflects a single-location bureaucratic environment. |

**Appendix Table A2. Illustrative recoding from interview, SWOT, and PEST material to hidden-risk blocks**

| **Source fragment / summary entry** | **Source type** | **First-cycle code** | **Second-cycle hidden-risk block** | **Interpretive value** |
| --- | --- | --- | --- | --- |
| "Reforms create opportunities, but resources are scarce; clearer rules are needed." | SWOT / interview | Regulatory ambiguity; resource constraints | Political-legal and institutional effects | Shows managerial demand for clearer rules and implementation capacity. |
| "There are too many formalities; they slow work down..." | SWOT / interview | Formalism; procedural overload | Organizational and managerial effects | Indicates administrative burden and friction in digital implementation. |
| "There is a real danger of data leakage." | SWOT / interview | Infrastructural vulnerability; data insecurity | Data and technologies | Captures dependence on integrated systems and security anxiety. |
| "Digital services make work more transparent, but sometimes they also slow it down." | SWOT / interview | Visibility / control; performance friction | Organizational and managerial effects | Illustrates the ambivalence of digitalization as both control and burden. |
| "It is not always safe to speak about violations..." | SWOT / interview | Weak contestability; internal pressure | Political-legal and institutional effects | Points to fragile accountability culture. |
| "Without connections it is difficult to move initiatives forward..." | SWOT / interview | Hierarchy; informal influence; low flexibility | Political-legal and institutional effects | Reveals persistence of vertical control and constrained initiative. |
| Opacity / "black box" motif in interview summaries | Interview / PEST | Opacity; low intelligibility | Explainability / opacity | Shows that explainability deficits are already normalized before full AI rollout. |
| Platform dependence, overload, and system fragility | PEST | Infrastructural dependence | Data and technologies | Connects broader digitalization perceptions to the environment into which AI is being introduced. |

**Appendix Table A3. Descriptive coding overview of the 53-interview analytic subset**

| **Hidden-risk block** | **First-cycle codes included** | **Number of coded segments** | **Number of interviews containing the theme** | **Notes on respondent distribution** |
| --- | --- | --- | --- | --- |
| **Political-legal and institutional effects** | Hierarchy; informal influence; need for clearer rules; weak trust in substantive accountability; limited flexibility; low safety of internal contestation; pressure not to speak about violations | **323** | **47** | Widely present across both groups, with especially dense articulation in staff narratives about hierarchy, safety of reporting, and informal influence, and repeated confirmation in managerial accounts of weak contestability and constrained initiative. |
| **Data and technologies** | Dependence on integrated systems; platform overload; infrastructural fragility; data-leakage anxiety; uneven technical preparedness; system dependence; insecurity of digital infrastructures | **169** | **51** | Present across both groups; especially visible in managerial accounts of cyber-risk and infrastructure design, and in younger and staff narratives about system overload, dependence, and data insecurity. |
| **Organizational and managerial effects** | Formalism; reporting burden; measurable-output pressure; objective-control motif; procedural friction; visibility pressure; digitalization as organizational load | **333** | **51** | The most densely coded block across the corpus. Especially prominent in staff interviews discussing workload, formalism, reporting pressure, and resource constraints, with repeated managerial confirmation of metricized control and organizational overload. |
| **Explainability / opacity** | Partial intelligibility; black-box experience; limited reconstruction of decision pathways; acceptance of system outputs; normalized opacity; weak everyday contestation | **79** | **36** | Present across respondent groups, but more often articulated indirectly through references to transparency, formal openness, visibility, and system objectivity than through explicit technical vocabulary of explainability. |
| **Market and environmental effects** | Centralized dependence; unequal technical capacity; infrastructural concentration; weak explicit articulation of competition concerns; weak explicit articulation of sustainability concerns | **22** | **20** | The least frequent block in the qualitative material. Where it appeared, it was expressed mainly through concerns about foreign IT dependence, data sovereignty, uneven technical control, and the concentration of infrastructural capacity rather than through explicit market-regulation or environmental language. |

**Note.** Counts were reconstructed from answer-level coded excerpts in the manager and staff interview matrices and cross-checked against the consolidated SWOT and PEST reports. The resulting totals correspond to the 53-interview analytic subset used in the article. “Number of coded segments” refers to the total number of answer-level excerpts assigned to a given second-cycle block; “Number of interviews containing the theme” refers to the number of unique interviews in which at least one such excerpt appeared. Because a single interview could contribute segments to more than one block, row totals are not mutually exclusive and do not sum to 53.
